# Preclinical activity of selinexor in combination with eribulin in uterine leiomyosarcoma

**DOI:** 10.1186/s40164-023-00443-w

**Published:** 2023-09-15

**Authors:** Sonam Mittal, Ishaque Pulikkal Kadamberi, Hua Chang, Feng Wang, Sudhir Kumar, Shirng-Wern Tsaih, Christopher J. Walker, Pradeep Chaluvally-Raghavan, John Charlson, Yosef Landesman, Sunila Pradeep

**Affiliations:** 1 Department of Obstetrics and Gynecology, Medical College of Wisconsin, Milwaukee, WI USA; 2 Karyopharm Therapeutics, Inc, Newton, MA USA; 3 Medical College of Wisconsin-Cancer Center, Milwaukee, WI USA; 4 Department of Physiology, Medical College of Wisconsin, Milwaukee, WI USA; 5 Medical Oncology, Medical College of Wisconsin, Milwaukee, WI 53226 USA

**Keywords:** LMS, ULMS, XPO1, Sarcoma, Eribulin, Selinexor, Doxorubicin

## Abstract

Leiomyosarcoma (LMS) is a rare soft tissue sarcoma (STS) that begins in smooth muscle tissue and most often initiates in the abdomen or uterus. Compared with other uterine cancers, uterine LMS (ULMS) is an aggressive tumor with poor prognosis and a high risk of recurrence and death, regardless of the stage at presentation. Selinexor is a first-in-class selective inhibitor of nuclear export (SINE) compound that reversibly binds to exportin 1 (XPO1), thereby reactivating tumor suppressor proteins and downregulating the expression of oncogenes and DNA damage repair (DDR) proteins. In this study, we evaluated the effects of selinexor in combination with doxorubicin and eribulin in the LMS tumor model in vitro and in vivo. Treatment of selinexor combined with eribulin showed synergistic effects on tumor growth inhibition in SK-UT1 LMS-derived xenografts. Immunohistochemical assessment of the tumor tissues showed a significantly reduced expression of proliferation (Ki67) and XPO1 markers following combination therapy compared to the control group. Global transcriptome analyses on tumor tissue revealed that the combination therapy regulates genes from several key cancer-related pathways that are differentially expressed in ULMS tumors. To our knowledge, this is the first preclinical study demonstrating the anti-cancer therapeutic potential of using a combination of selinexor and eribulin in vivo. Results from this study further warrant clinical testing a combination of chemotherapy agents with selinexor to reduce the morbidity and mortality from ULMS.

**Supplementary Information:**

The online version contains supplementary material available at 10.1186/s40164-023-00443-w.

## To the editor

Uterine leiomyosarcoma (ULMS) is a rare, malignant, mesenchymal tumor of smooth muscle with its incidence being nearly 1% of all uterine malignancies. It comprises ~ 70% of all uterine sarcomas and contributes to a large proportion of uterine cancer deaths [1]. The prognosis of ULMS is poor, with overall 5-year survival rate of ULMS patients ranging to only 40–60% [2]. ULMS is commonly insensitive to chemotherapy and surgical resection is the mainstay treatment [3]. So far, doxorubicin, and gemcitabine/docetaxel remain the most effective treatment regimens for ULMS patients with recurrent and advanced disease but often associated with toxicity [4]. This emphasizes the urgent need for development of novel therapies.

Exportin-1 (XPO1) is a nuclear transport protein that shuttles various cell regulatory proteins from the nucleus to the cytoplasm [5]. Selinexor, a selective inhibitor of nuclear export (SINE) agent, blocks XPO1 function and induces nuclear localization of tumor suppressor and growth regulatory proteins, including p21, p27, p53, p73, STAT3, BRCA1, FOXO, CDKN1A, RB1, IkB, APC, NPM1, and survivin [6]. Ultimately, the nuclear enrichment or sequestering of these cargo proteins leads to a reduction of oncogenic translation and the activation of cell cycle arrest, thereby initiating cancer cell death. Its efficacy in patients with diffuse large B-cell lymphoma and multiple myeloma has led to FDA approval [7]. In this current study, the therapeutic potential of selinexor was examined against ULMS both in cell culture and in a murine xenograft model.

Selinexor, combined with DNA-damaging agents (DDAs), doxorubicin and eribulin, inhibited cell proliferation, including clonogenic growth, and induced apoptosis of ULMS cells in vitro and the growth of ULMS xenografts (see Additional file 1: Methods).

**Fig. 1:**
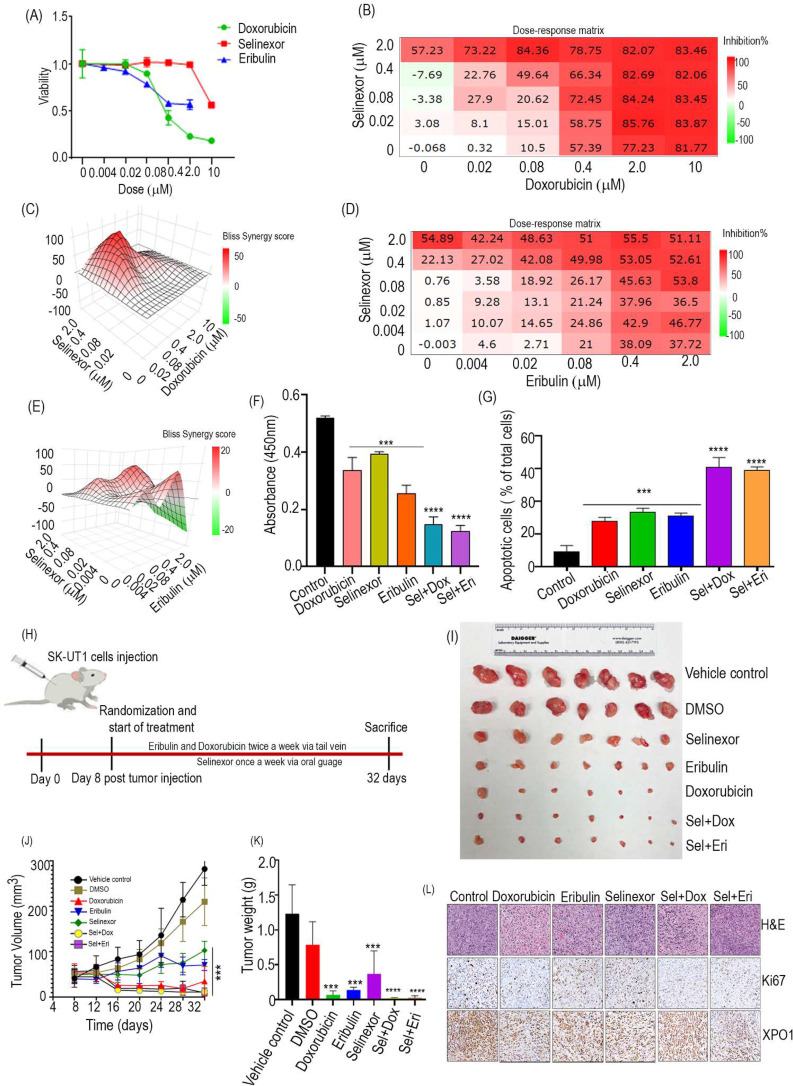
Combination effects of selinexor with chemotherapeutics. **(A)** Cell viability of SK-UT1 cell line treated with increasing concentrations of selinexor, eribulin and doxorubicin for 72 h. SK-UT1 cells were treated with selinexor (dose range 0.02 to 2µM) in combination with either doxorubicin (dose range 0.02 to10µM), eribulin (dose range 0.004 to 2µM) as a 5 × 5 or 6 × 6 matrix of concentrations in a cell viability assay. **(B)** Dose-response plots of Selinexor + doxorubicin. **(C)** Bliss synergy plots of Selinexor + doxorubicin. **(D)** Dose response plots of selinexor + eribulin. **(E)** Bliss synergy plots of selinexor + eribiulin. **(F)** Colony formation assay in SK-UT1 cells pretreated with drug as indicated for 2 weeks. **(G)** Apoptosis evaluation after 72 h of single-agent or combined treatment in SK-UT1 cells. 20nM selinexor, 80nM doxorubicin, 20nM. Statistical analysis was done using one-way ANOVA, and asterisks show significant differences (***P < 0.001; ****P < 0.0001). **(H)** Schematic of the treatment protocol. **(I)** Photographs of excised tumors in each group **(J** and **K)** The average tumor volume and tumor weight of SK-UT1 xenografted nude mice treated with selinexor (15 mg/kg), eribulin(1 mg/kg) and doxorubicin(4 mg/kg) either alone or in combination, for 32 days. Results are represented as the mean ± SEM of at least seven animals in each group (*P < 0.05). **(L)** Formalin-fixed tumor tissues were immunostained with Ki67 and XPO1 antibodies (n = 3 animals/group)

Previous studies have shown that TP53 is the most commonly mutated gene in leiomyosarcoma and P53 mutations are associated with longer progression free survival on eribulin [8, 9]. Having demonstrated that P53 mutations significantly reduced the anti-lymphoma efficacy of selinexor and presence of Mut-TP53/p53 + expression predicts resistance to selinexor, we sought to determine the effects of selinexor in combination with eribulin and doxorubicin on two different ULMS cell lines that included SK-UT1 (p53-mut) and SK-UT1-B (p53-WT) [10]. Both the cells were treated with various concentrations of selinexor, eribulin and doxorubicin. Sensitivity was evaluated by calculating the IC50 using nonlinear regression. The sensitivities among the cell lines varied. SK-UT1-B cells were more sensitive to selinexor, doxorubicin, and eribulin than SK-UT1 cells. Expression of Mut-p53 in SK-UT1 cells confers resistance to selinexor treatment (Fig. 1A, SK-UT1 cells and Figure S1A, SK-UT1-B cells). Therefore, we investigated synergistic anti-cancer activity of selinexor in combination with doxorubicin and eribulin. Synergistic effects were found in SK-UT1 cell lines when selinexor was combined with doxorubicin and eribulin (Fig. 1B-G; Figure S2A, B). SK-UT1-B cells did not show any synergism in all the combination treatments; selinexor and doxorubicin (Figure S1B-F), selinexor and eribulin (Figure S1G-K; S2C, D).

To further expand on the in vitro observations, mice engrafted with the SK-UT1 cells were treated with vehicle, selinexor, doxorubicin, eribulin, or selinexor + doxorubicin or selinexor + eribulin. Selinexor, combined with doxorubicin, showed a significant reduction in tumor growth compared to selinexor alone. However, doxorubicin alone or selinexor + doxorubic in treatment has similar effect on tumor growth because doxorubicin alone at 4 mg/kg resulted in a greater tumor regression. As selinexor + doxorubic treatment showed more apoptotic cell death during in vitro experiment (Figure S2A, B), lowering the concentration of doxorubicin in tumor bearing mice could potentially lead to a synergistic effect when combined with selinexor. The combination of selinexor with eribulin had a synergistic anti-cancer effect on reducing primary tumor volumes of SK-UT1 xenografts, which was associated with their decrease in XPO1 expression and reduced proliferation (Ki67) (Fig. 1H-L). In this experiment, both monotherapy and combination regimens had no adverse effect on the body weight of the animals (Figure S3A, B). Taken together, our results demonstrate a potent synergistic effect of selinexor when combined with eribulin to treat ULMS.

**Fig. 2:**
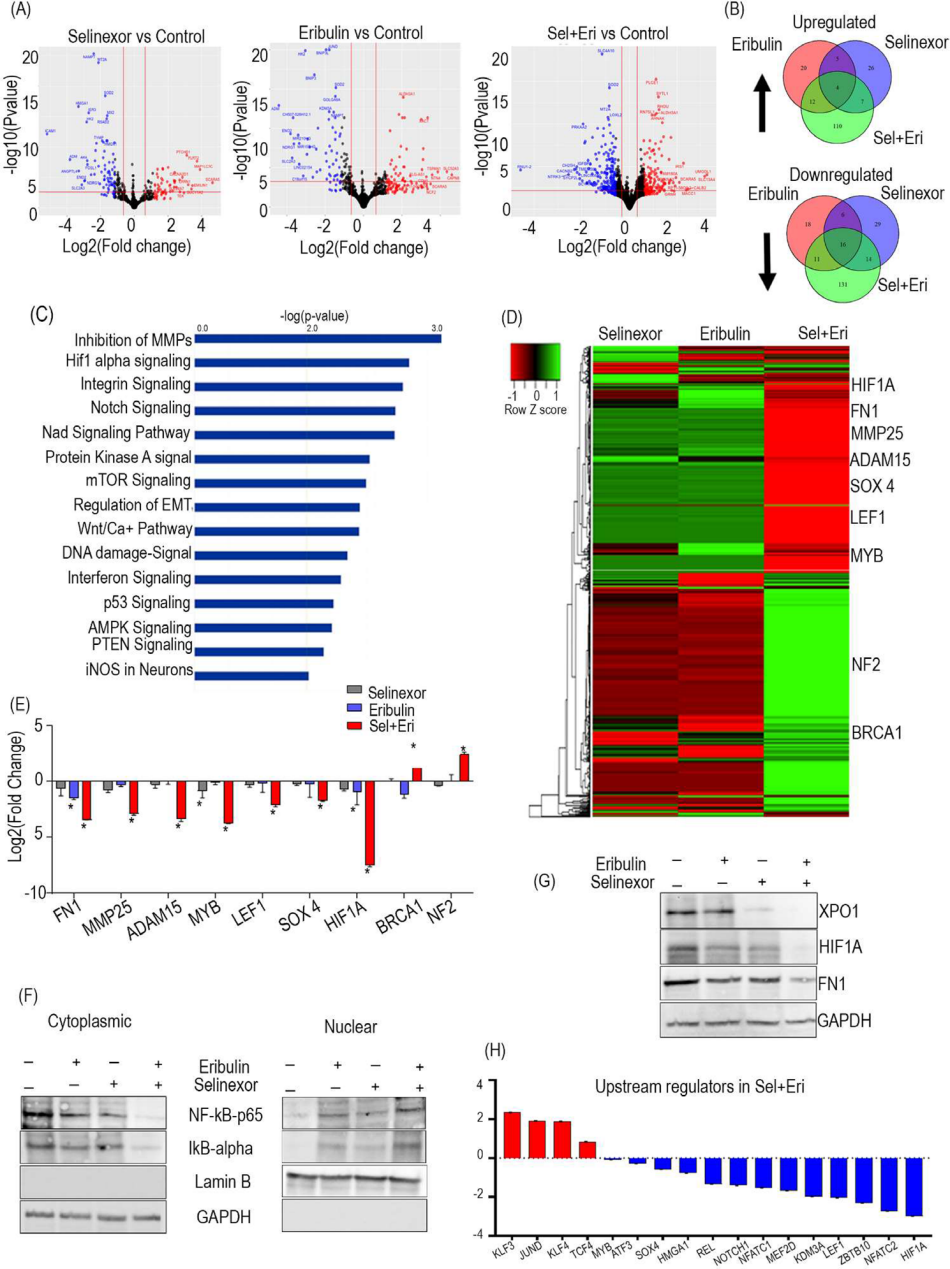
Transcriptome analyses of SK-UT1 cells xenografts tissue from selinexor, eribulin, and selinexor + eribulin treatment groups. **(A)** The volcano plot of the DEGs obtained from global transcriptome analyses in each treatment group relative to vehicle-treated SK-UT1 tumor is shown (n = 3/group). **(B)** Venn diagrams representing the overlap of DEGs among different treatment groups. The upregulated and downregulated genes were analyzed and represented separately. **(C)** Top canonical pathways enriched by differentially expressed genes by Ingenuity Pathway Analysis (IPA). Pathways were ranked based on p-value, where the bars represent the inverse log of the p-value (x-axis). A p-value < 0.05 by Fisher’s exact test was considered to select statistically significant pathway annotation. **(D)**. Comparative heatmap of the differentially expressed genes in different treatment groups, as determined by heat mapper. **(E)** Validation of selected genes that showed significant differential expression following selinexor + eribulin combination treatment. qPCR validation of selected cancer-related genes obtained from RNA-sequencing. Results are represented as the mean ± SEM. (*P < 0.05; n = 3/group). **(F)** Western blotting validation of selected cancer-related genes. **(G)** Selinexor treatment showed minimal nuclear entrapment of IκB-α, but combined treatment with selinexor and eribulin further increased nuclear retention of IκB-α **(H)** Bar graphs show the IPA tool predicted a list of significantly activated and inhibited upstream transcription regulators in each treatment group. A z-score greater than 2.0 defines significant activation of the node, whereas a z-score less than 2.0 defines inhibition. HIF1A, MYB and SOX4 are several transcription regulators predicted to be inhibited by selinexor + eribulin treatment

To examine the molecular mechanism underlying the enhanced anti-cancer potential of the combination therapy, we performed RNA sequencing transcriptome analyses on SK-UT1 cells xenograft tumor. Our study demonstrates that in mono and combination treatment groups, differentially expressed genes (DEGs) were primarily enriched in the apoptosis, hypoxia, EMT, and TNFa signaling via NFkB pathways (Fig. 2A, B; Figure S4A-C). Using ingenuity pathway analysis (IPA), we then performed comparative pathway enrichment analyses between DEGs affected by selinexor alone or in combination with eribulin. We found that the DEGs in combination treatment are enriched in cancer-related pathways like inhibition of matrix metalloproteinases (MMPs), HIF1A signaling, and regulation of epithelial to mesenchymal transition (EMT). Compared to the monotherapy treatment, the selinexor + eribulin treatment showed enhanced anti-cancer effects by reducing the expression of genes related to EMT transition, angiogenesis, and metastasis like ADAM15, HIF1A, FN1, and SOX4, and upregulating tumor suppressors genes like NF2, and BRCA1 (Fig. 2C-E). We also validated the expression of some of these genes using western blotting (Fig. 2F). Previous study has shown that NFκB transcriptional activity is upregulated in cells that are naturally resistant or have acquired resistance to selinexor [11]. IκB-α, also known as the inhibitor of NF-κB, serves as a cargo for XPO1. It inhibits NF-κB transcription factor by blocking it in an inactive state within the cytoplasm. This action effectively prevents NF-κB from translocating into the nucleus and binding to DNA [12]. However, higher expression of XPO1 induce excessive export of IκB-α from the nucleus to the cytoplasm. In the cytoplasmic compartment, IκB-α undergoes inactivation through proteasome-mediated degradation. This series of events leads to an increase in NF-κB’s transcriptional activity, promoting inflammation and the development of tumors. In line with these findings, we sought to determine if combination treatment could overcome resistance to selinexor in SK-UT1 cells by preventing IκB-α degradation. Nuclear-cytoplasmic fractionation of SK-UT1 cells treated with selinexor, eribulin or their combination confirms the enhanced nuclear localization of IκB-α and reduced NF-κB nuclear transport (Fig. 2G). This study addresses the mechanism of apoptosis that occurs independently of the p53 protein in SK-UT1 cells with mutant p53. Apotosis in these cells are enhanced by combined treatment of selinexor and eribulin, which lead to elevated nuclear accumulation of IκBα (Figure S2B).

To further identify the potential upstream regulators that mediate the gene expression changes in RNA-Seq, an upstream regulator analysis (URA) was performed using IPA tool. The upstream transcription regulators activated in combination were JUND1, KLF3, KLF4, and TCF4, which affect a diverse array of target genes. For example, KLF4 activation might repress FN1 expression, whereas JUND1 activation might down-regulate HIF1A, SOX4, and several other genes, as seen in the RNA-Seq data. We also found a reduction in some of the well-known cancer-related transcription factors like MYB, and HIF1A whose downstream target genes are shown in Figure S5A, and B. The mechanistic network analyses suggest that the inhibition of MYB possibly mediates HIF1A down-regulation, which, in turn, affects various downstream oncogenic factors, as shown in Figure S5C. These observations indicate that the selinexor + eribulin treatment alters the expression of crucial transcription factors that mediate the downstream changes in the expression of a vast array of genes in the SK-UT1 tumor transcriptome. Our findings have provided a molecular rationale for combining these agents in the clinical setting.

### Electronic supplementary material

Below is the link to the electronic supplementary material.

Supplementary Material 1.

## Data Availability

All relevant data are presented in the paper or included as supplementary files. Raw data generated in this study are available upon reasonable request from the corresponding authors.

## List of abbreviations

SINE: Selective inhibitor of nuclear export
XPO1: Exportin 1
DDR: DNA damage repair
ULMS: Uterine leiomyosarcoma
LMS: Leiomyosarcomas
STS: Soft-tissue sarcomas
DDAs: DNA damaging agents
DEGs: Differentially expressed genes
IPA: Ingenuity pathway analysis
MMPs: Matrix metalloproteinases
EMT: Epithelial to mesenchymal transition
URA: Upstream regulator analysis
