# Supplementary material for: Preclinical activity of selinexor in combination with eribulin in uterine leiomyosarcoma
Source: Exp Hematol Oncol. 2023 Sep 15;12:78. doi: 10.1186/s40164-023-00443-w (PMC10503035; doi:10.1186/s40164-023-00443-w)
Supplement: Supplementary file 1 — Supplementary Material 1. [file 40164_2023_443_MOESM1_ESM.docx]

**Material and Methods**

**Cell culture**

Human uterine leiomyosarcoma cell lines SK-UT1 (p53-mut) and SK-UT1-B (p53-WT) cell lines were obtained from American Type Culture Collection (ATCC, Gaithersburg, MD, USA) and cultured following the manufacturer's recommendations. Both the cell lines were cultured in MEM (Sigma-Aldrich, Saint Louis, MO, USA) in a humidified atmosphere of 5% CO_2_ at 37 °C. Media were supplemented with 10% fetal bovine serum (FBS, Omega Scientific, Inc, USA), penicillin (100 U/ml) and streptomycin (0.1 mg/ml).

**Reagent and antibodies**

Selinexor (KPT-330) and Pluronic F-68 were obtained from Karyopharm Therapeutics (Newton, MA, USA). For *in vitro* administration, selinexor was dissolved in dimethyl sulfoxide (DMSO, Sigma-Aldrich) to a concentration of 10 mM. For *in vivo* administration, selinexor was dissolved in 0.6% w/v aqueous Pluronic F-68 (Karyopharm Therapeutics, Newton, MA, USA). Doxorubicin (Cat N0-15005, Cayman Chemical, MI, USA) and eribulin (Cat No-HY-13442A, MedChemExpress LLC, NJ, USA) were dissolved in DMSO to a concentration of 10mM and stored at −20°C. Antibodies for IHC include XPO1/CRM1 (Bethyl Laboratories Inc., Montgomery, TX, USA, A300-469A, 1:15k), and Ki67 (Biocare Medical, Pacheco, CA, USA, Prediluted).

**Cell viability assay and determination of drug synergy**

SK-UT1 and SK-UT1-B cells (5 × 10^3^ cells per well) were seeded into 96-well plates in 100 µl of complete growth medium and then treated with the indicated drugs in triplicate. Cell viability was tested using the cell counting kit-8 (CCK-8) assay (Dojindo Molecular Technologies, Japan, CK04) according to the manufacturer's instructions. The synergistic anti-cancer activity of selinexor in combination with eribulin and doxorubicin in SK-UT1 and SK-UT1-B was investigated by Bliss independence analysis using Bliss Independence and Highest Single Agent (HSA) methods, using the Synergy Finder 2.0 software. The Excess over Bliss score = 0 indicates that the combination treatment is additive. The Excess over Bliss scores > 0 indicates activity greater than additive (synergy). The Excess over Bliss scores < 0 indicates the combination is less than additive (antagonism).

**Colony formation assays**

Colony formation assay was applied to characterize the 2-dimensional clonogenic growth of sarcoma cells after treatment with cytotoxic agents. Therefore, SK-UT1 and SK-UT1B cells were plated in a 6-well plate at very low density (500 cells per 2 mL cell culture medium per well), allowing each single cell to grow into a colony. After 48 hours, cells were incubated with selinexor, doxorubicin, eribulin, or selinexor+doxorubicin and selinexor+eribulin containing medium at the indicated concentrations. After 2 weeks, cells were rinsed with 1X PBS, fixed in 5% glutaraldehyde for 20 min, and stained with 0.5% crystal violet (Sigma Aldrich, St. Louis, MO, USA) for 20 min. Plates were washed with water and dried before scanning. Crystal Violet was solubilized with 10% acetic acid, and absorbance was measured at 450 nm in a microplate reader (Tecan, Mannedorf, Switzerland). All experiments were performed in triplicates. Statistical analysis was performed using one-way ANOVA with Prism 9.5.0 (GraphPad Software, CA, USA).

**Annexin V and propidium iodide (Annexin V-PI) apoptosis analysis**

Annexin V-PI staining was performed using flow cytometric analysis as previously described. Briefly, 1 × 10^6^ SK-UT1 cells were cultured and treated with selinexor, doxorubicin, eribulin, or selinexor+doxorubicin and selinexor+eribulin containing medium at the indicated concentrations for 72 hours and digested with free trypsin. Staining was performed using Apoptosis Detection Kit II (BD Biosciences, USA). Cells were harvested and washed twice with 1X PBS. Cells were suspended in 1X binding buffer containing 5 µl of FITC conjugated Annexin V and 5 µl. of PI for 30 min in the dark. The samples were analyzed using LSR-II flow cytometer (BD, San Jose, CA, USA), and the data were analyzed by FlowJo v10 software.

***In vivo* xenograft model of leiomyosarcoma**

For *in vivo* experimental purposes, 4-6 weeks old female athymic nude mice (Athymic Nude-Foxn1nu) were purchased from Envigo and housed in pathogen-free conditions. All experiments on mice were performed in accordance with the Medical College of Wisconsin Institutional Animal Care and Use Committee (IACUC). Animal health was monitored daily.

For subcutaneous tumor cell inoculation, SK-UT1 cells were trypsinized, washed, and resuspended in Ca^2+^ and Mg^2+^ free 1X Hanks' balanced salt solution (HBSS, GIBCO, Carlsbad, CA, USA) and 1×10^6^ cells were injected subcutaneously into the left flank region. Tumor-bearing mice were randomly divided into seven groups (n = 7/group) after tumors had grown to an average of 100 mm^3^. Mice were treated with Vehicle control, selinexor (15 mg/kg) via oral gavage, DMSO control, eribulin (1mg/kg), and doxorubicin (4mg/kg) by intravenous injection either alone or in combination. Tumor volume was calculated using V = (length × width×width) ½. once any mice became moribund, the experiment was terminated, and tumors were harvested. Subcutaneous tumor measurement was performed weekly in mice exhibiting palpable subcutaneous tumors until humane endpoints. Tumor tissue was fixed in 10% formalin for immunohistochemistry (IHC) and RNAseq. Briefly, four-micron sections were stained with hematoxylin & eosin (H&E, Richard-Allen Scientific) or used for IHC. Sections were deparaffinized and rehydrated, placed in Declare working buffer, steam-cooked for antigen retrieval, cooled, transferred to 3% hydrogen peroxide, blocked, incubated with primary antibodies, then secondary antibodies (Cell Marque Hi-Def Polymer Amplifier). Slides were stained with DAB and hematoxylin, dehydrated, mounted, then cover-slipped. Images were taken with an Aperio AT Turbo slide scanner at 20X.

**RNA sequencing and transcriptome analysis**

Ribodepleted RNAseq was performed by HudsonAlpha Discovery Sequencing and Bioinformatics Division (Huntsville, AL, USA). Briefly, RNA was extracted from FFPE slides using the Covaris truXTRAC FFPE total NA Plus Kit according to manufacturer's recommendation, run on RNA Fragment Analyzer chips, and assessed with Ribogreen Assays. Suitable RNA extractions were prepared using Illumina Ribo Zero Plus Ligation Library Prep kits, then evaluated with Kapa qPCR kits. Sequencing was performed on Illumina Novaseq instruments.

RNAseq reads were examined with fastqc, trimmed with trimgalore, aligned with STAR, then gene-level counts were calculated using the htseq. Gene expression normalization and comparisons between indicated groups were performed with DeSeq2. As shown, pathway analysis was done using ingenuity pathway analysis and Gene Set Enrichment Analysis with Hallmark Molecular Signatures Database.

**Quantitative real time-PCR (qRT-PCR)**

Total RNA was isolated from the cells using the RNeasy Mini Kit (Qiagen, Valencia, CA, USA), and first-strand cDNA was transcribed using iScript reverse transcription supermix (Biorad, Hercules, CA, USA). qRT-PCR was performed using CFX Connect Real-Time PCR systems (Biorad, Hercules, CA, USA) and SYBR Premix Ex Taq II (Biorad, Hercules, CA, USA).

**Westen Blotting**

For western blot analysis, cells were lysed using 1x RIPA lysis buffer containing freshly added protease inhibitor cocktails (Thermo Fisher Scientific Inc., Rockford, IL, USA). The protein concentration was determined by Bradford assay; 20 µg of total protein from each sample was resolved using 10 % SDS-PAGE followed by western blot analysis. Blocking of non-specific proteins was performed by incubating the blots in 5% skimmed milk in 1X TBST for 1 h at room temperature. The blots were incubated with anti-bodies against XPO-1 (cat. no. 27917-1-AP Proteintech), IkB-alpha (cat. no. 10268-1-AP, Proteintech), Lamin B1 (cat. no. 13435S,), GAPDH (cat. no. 2118L), NF-kB-p95 (cat. no. 8242T), FN1 (cat. no. 26836S), and HIF1-alpha (cat. No. 36169S) all from cell signaling technology Inc. Protein expression was detected using HRP-conjugated anti-rabbit (cat. no. 7074, cell signaling technology Inc.). Protein bands were visualized using ECL substrate (cat. no. 34580 Thermo Fisher Scientific Inc., Waltham, MA).

**Statistical analysis**

All statistical analysis was performed in GraphPad Prism software (9.5.0) For all *in vitro* experiments, three or six technical replicates were analyzed for each experiment, and results are presented as the mean ± S.E.M. of three biological replicates. Quantitative analyses were carried out using unpaired two-tailed Student’s t test with equal standard deviation. Statistical analyses of *in vivo* experiments were performed with one-way NOVA followed by Dunnett’s multiple comparison test. P-values <0.05 (*), <0.01 (**), <0.001 (***), and <0.0001 (****) were considered as statistically significant.

**Supplementary Table**

Table-S1 Real-time PCR primers used in this study.

| FN1 F | ACAACACCGAGGTGACTGAGAC |
| --- | --- |
| FN1 R | GGACACAACGATGCTTCCTGAG |
| MMP25 F | TGACAAGCCCACAAGGAAACCC |
| MMP25 R | GATGGCGTCAAAATTGCCCTCAC |
| ADAM15 F | CAAACTGTGCTGAAGGACCATGC |
| ADAM15 R | GTGGTTTTCTGGGCATGATGCAG |
| SOX4 F | GACATGCACAACGCCGAGATCT |
| SOX4 R | GTAGTCAGCCATGTGCTTGAGG |
| LEF1 F | CTACCCATCCTCACTGTCAGTC |
| LEF1R | GGATGTTCCTGTTTGACCTGAGG |
| MYB F | GGGAACAGATGGGCAGAAATCG |
| MYB R | GCTGGCTTTTGAAGACTCCTGC |
| HIF1A F | TATGAGCCAGAAGAACTTTTAGGC |
| HIF1A R | CACCTCTTTTGGCAAGCATCCTG |
| NF2 F | TGAACGCACGAGGGATGAGTTG |
| NF2 R | GCCTTTTCAGCCAACAGGTCAG |
| BRCA1 F | CTGAAGACTGCTCAGGGCTATC |
| BRCA1 R | AGGGTAGCTGTTAGAAGGCTGG |

**Supplementary Figures**


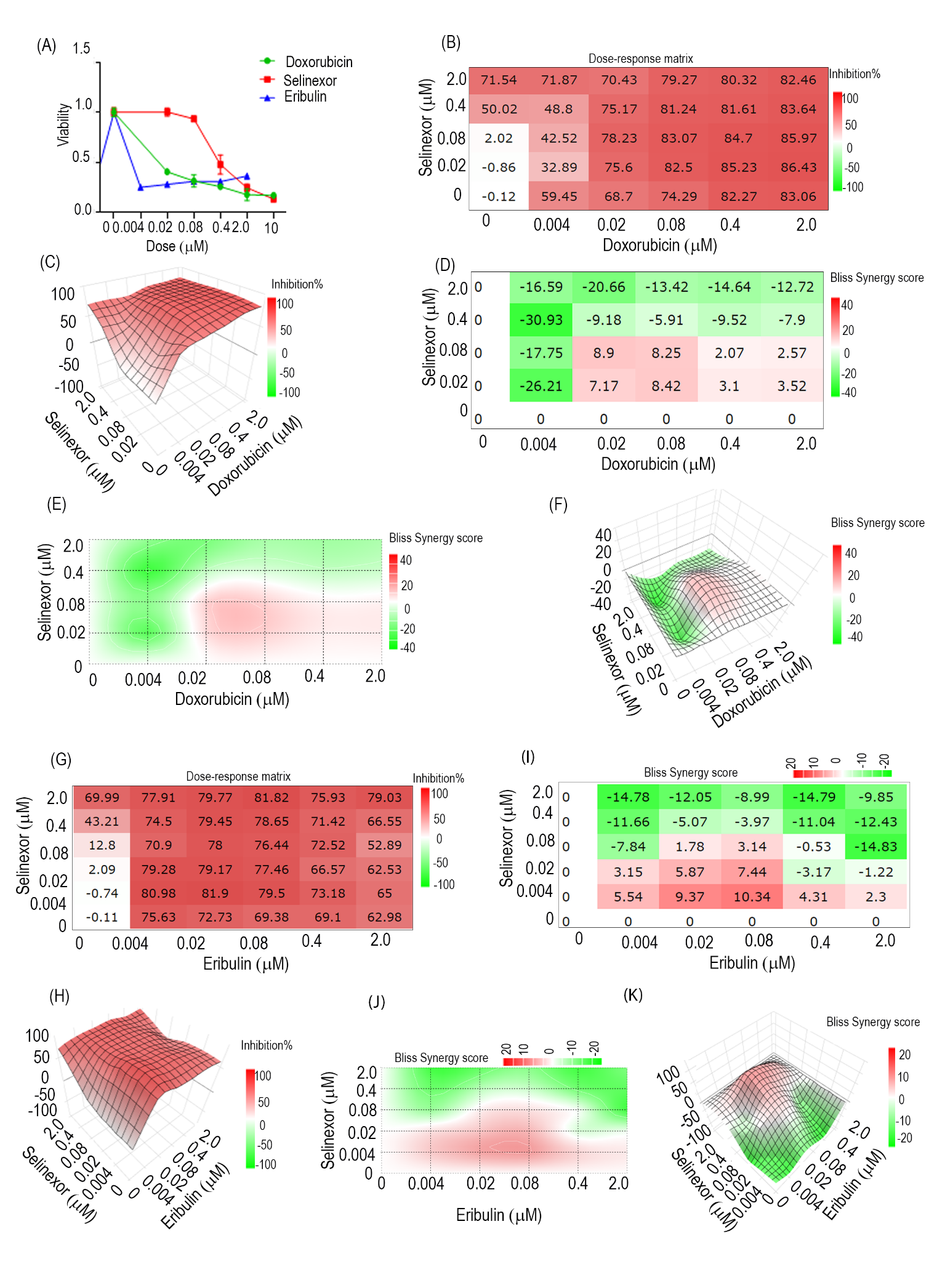


Sup Fig-1

**Supplementary Figure- 1**

Combination effects of selinexor with chemotherapeutics. (A) Cell viability of SK-UT1-B cell line treated with increasing concentrations of selinexor, eribulin and doxorubicin for 72 hours. SK-UT1-B cells were treated with selinexor (dose range 0.02 to 2μM) in combination with either doxorubicin (dose range 0.02 to10μM), eribulin (dose range 0.004 to 2μM) as a 5 × 5 or 6 × 6 matrix of concentrations in a cell viability assay. (B and C) Dose-response plots of selinexor + doxorubicin. (D-F) Bliss synergy plots of selinexor+ doxorubicin. (G and H) Dose response plots of selinexor +eribulin. (I-K) Bliss synergy plots of selinexor+ eribiulin.


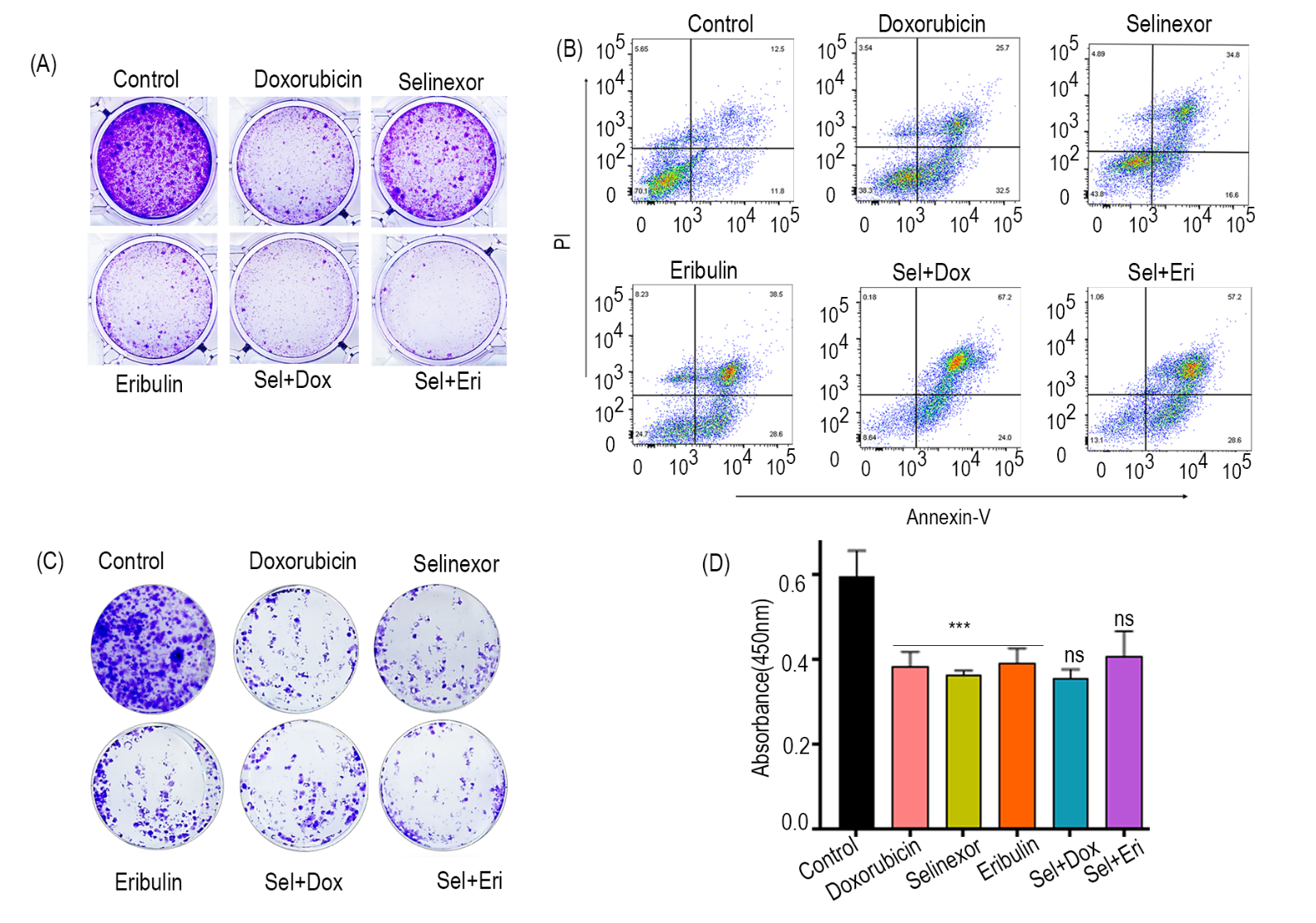


Sup Fig-2

**Supplementary Figure-2**

The synergistic effect of selinexor with doxorubicin and eribulin in SK-UT1 and SK-UT1-B cells (A) Colony formation assay in SK-UT1 cells pretreated with drug as indicated for 2 weeks. (B) Apoptosis evaluation after 72 hours of single-agent or combined treatment in SK-UT1 cells. 20nM selinexor, 80nM doxorubicin, 20nM eribulin. (C and D) Colony formation assay in SK-UT1-B cells pretreated with drug as indicated for 2 weeks Statistical analysis was done using one-way ANOVA, and asterisks show significant differences (***P < 0.001).


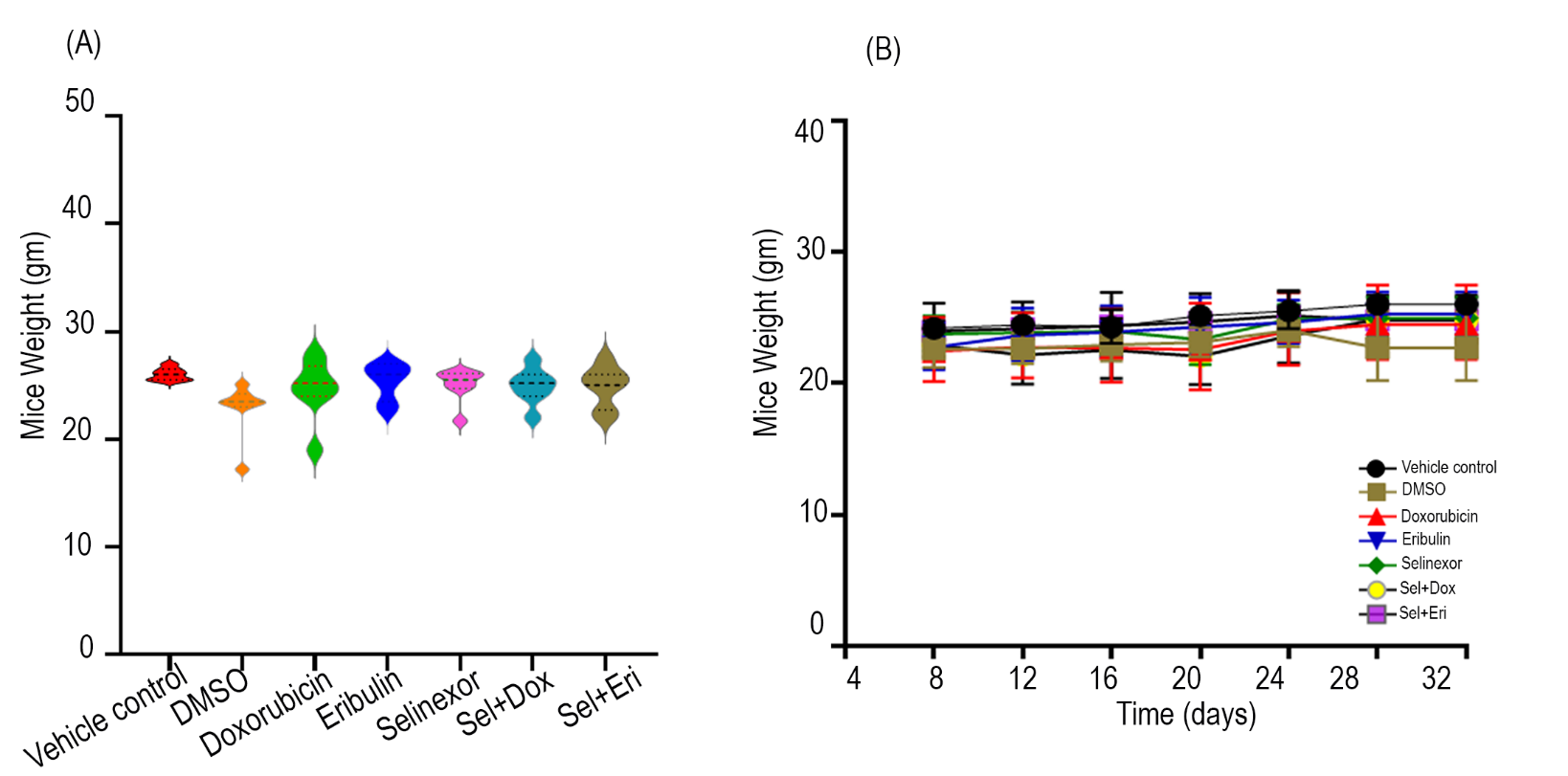


Sup Fig-3

**Supplementary Figure-3**

Effect of selinexor (15 mg/kg), eribulin (1mg/kg), doxorubicin (4mg/kg), and the combination on body weight of mice bearing SK-UT1 cells at (A) the end of the experiment and (B) throughout the treatment period. Statistical analysis was done using one-way ANOVA and the differences in body weight were non-significant in all the treatment conditions.


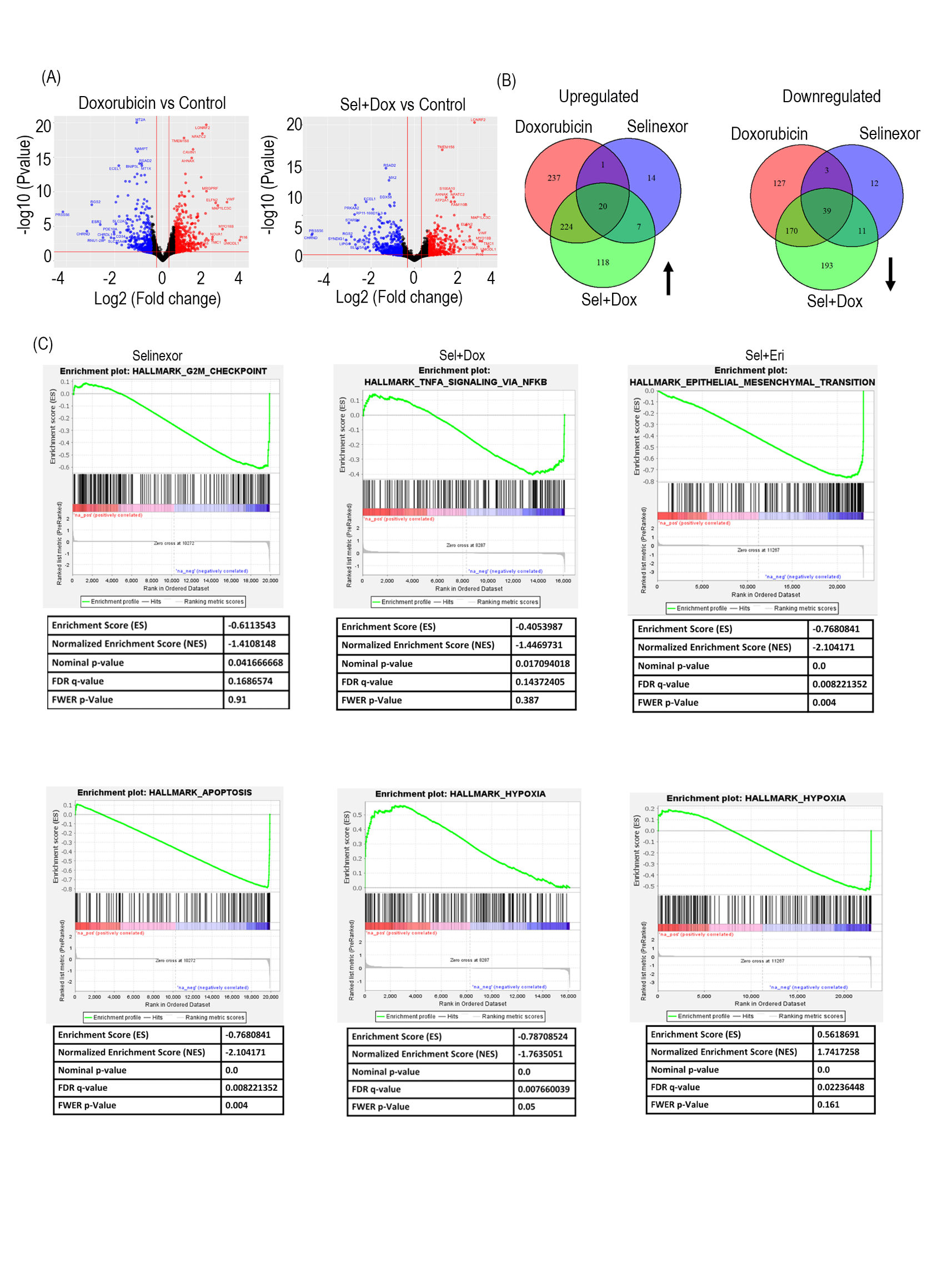


Sup Fig-4

**Supplementary Figure-4**

A) Volcano plots show differentially expressed genes between doxorubicin vs. control and selinexor+doxorubicin vs. control treated xenografts. (B) Venn diagrams of up-and down-regulated genes depicted in panel A. (C) GSEA plots of enriched signatures in tumor tissue excised from SK-UT1 cells with (left) selinexor vs. vehicle (middle) selinexor+doxorubicin vs. doxorubicin, and (right) selinexor+eribulin vs. eribulin. An FDR q-value < 0.05 was considered statistically significant.


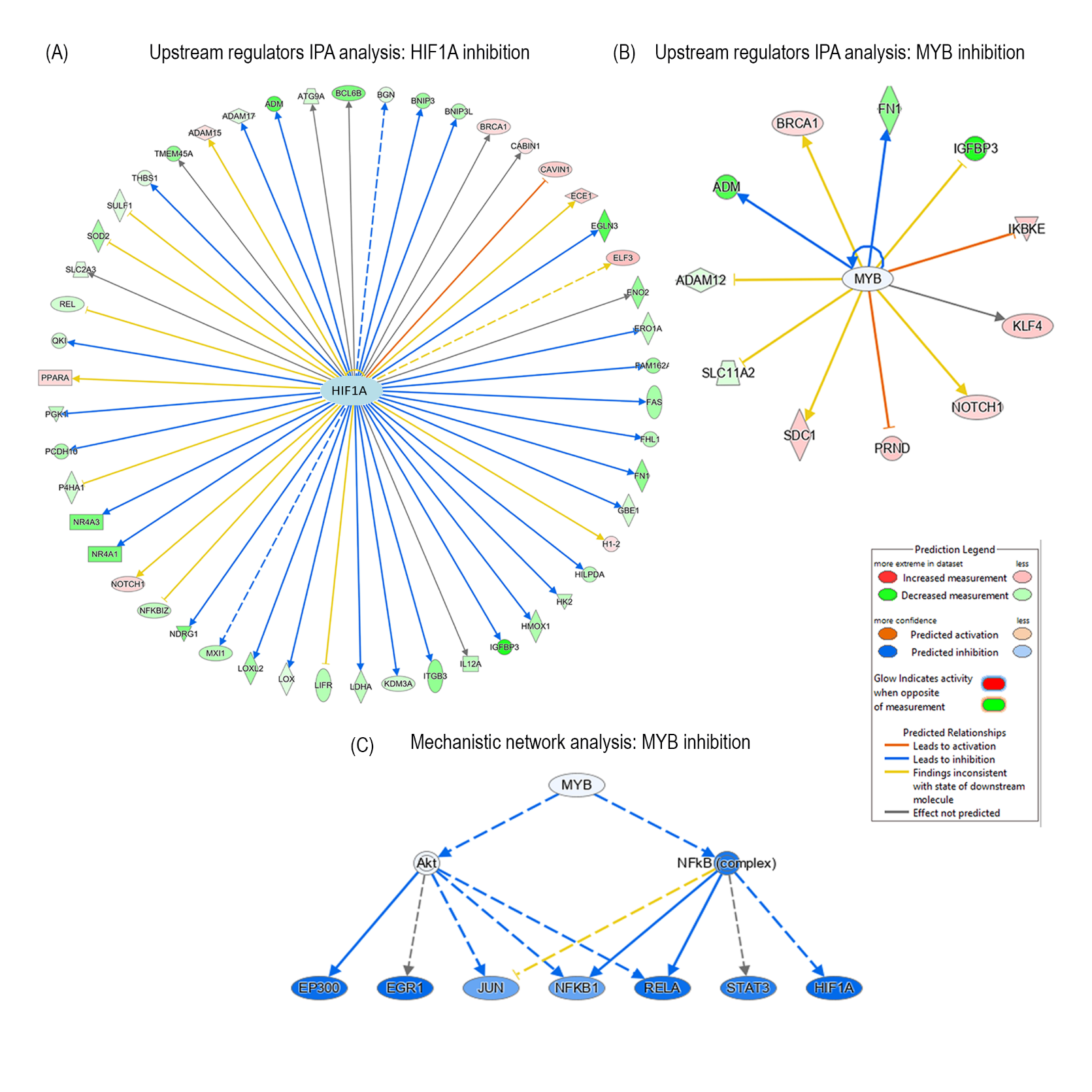


Sup Fig-5

**Supplementary Figure-5**

(A and B) Target molecules of HIF1A and MYB from the list of DEGs upon selinexor+eribulin treatment are shown. (C) Mechanistic pathway analyses of MYB, according to the IPA knowledge base, establish the network of molecular targets that are possibly affected by the combination treatment.
